# Can Circulating Cell-Free DNA or Circulating Tumor DNA Be a Promising Marker in Ovarian Cancer?

**DOI:** 10.1155/2021/6627241

**Published:** 2021-04-12

**Authors:** Ming Yu, Yu Zhu, Lichen Teng, Jialin Cui, Yajuan Su

**Affiliations:** Department of Clinical Laboratory, Harbin Medical University Cancer Hospital, Harbin, China

## Abstract

In recent years, the studies on ovarian cancer have made great progress, but the morbidity and mortality of patients with ovarian cancer are still very high. Due to the lack of effective early screening and detecting tools, 70% of ovarian cancer patients are diagnosed at an advanced stage. The overall survival rate of ovarian cancer patients treated with surgical combined with chemotherapy has not been significantly improved, and they usually relapse or resist chemotherapy. Therefore, a novel tumor marker is beneficial for the diagnosis and prognosis of patients with ovarian cancer. As the index of “liquid biopsy,” circulating cell-free DNA/circulating tumor DNA (cfDNA/ctDNA) has attracted a lot of attention. It has more remarkable advantages than traditional methods and gives a wide range of clinical applications in kinds of solid tumors. This review attempts to illuminate the important value of cfDNA/ctDNA in ovarian cancer, including diagnosis, monitoring, and prognosis. Meanwhile, we will present future directions and challenges for detection of cfDNA/ctDNA.

## 1. Introduction

Ovarian cancer is the most lethal malignancy of female reproductive system, while epithelial ovarian cancer is the most common type. Although the incidence of ovarian cancer is less than cervical cancer and uterine body cancer, the death rate of ovarian cancer ranks first in gynecological tumors, which generates a threat to women's health and life. Because of the complicated characteristics of ovarian cancer and the tumor being just located in the pelvic cavity, early ovarian cancer patients often have no obvious symptoms and signs; as a result, only about 25% patients can be diagnosed before they get worse [1]. The main treatment principle of ovarian cancer is surgery, supplemented of chemotherapy. Surgery is the preferred option for ovarian cancer, which can stage tumors, develop treatment plan, and judge the prognosis. Chemotherapy also contributes to the treatment of ovarian cancer; it is divided into neoadjuvant chemotherapy, postoperative chemotherapy, and postrelapse chemotherapy. For most patients, the main chemotherapy regimen is a combination of platinum and paclitaxel [2]. In addition, there are targeted radiotherapy and immunotherapy; advances have been made in the clinical treatment of ovarian cancer with bevacizumab (a recombinant human monoclonal IgG1 antibody that acts by inhibiting the biological activity of human vascular endothelial growth factor) or the poly ADP-ribose polymerase (PARP) inhibitor olaparib [3]. Although they have initial treatment response and are sensitive to chemotherapy, most of them tend to recur and produce resistance to chemotherapy drugs [4]; the 5-year survival rate is less than 30%. Therefore, early diagnosis is very important to monitor therapy response and improve prognosis of patients.

Imaging examination and serum tumor markers are widely employed as diagnostic technologies in clinical detection of ovarian cancer; unfortunately, these methods have not researched the standards of high sensitivity and specificity for early diagnosis; mortality did not significantly differ between screened women and those with no screening [5]. Transvaginal ultrasound has limited ability to distinguish between benign and malignant lesions, and it is difficult to find small tumors [6]. The detection of serum cancer antigen 125 (CA125) has low sensitivity, which makes it difficult to detect early lesions. Meanwhile, it also has poor specificity, because it can be detected in other nonmalignant diseases, which is likely to result in false positive [7]. Histopathological biopsy [8] is also one of the diagnostic tools for ovarian cancer, which has been regarded as the gold standard. However, it is time-consuming and costly; in addition, there is difficulty in sampling and it can make patients painful and risky; moreover, the tissue samples cannot be applied repeatedly. Based on the above, it is particularly necessary to find a non-invasive, repeatedly, early tumor marker with high sensitivity and specificity for detection and diagnosis of ovarian cancer.

The detection of cfDNA/ctDNA is called “liquid biopsy,” which is an emerging technology. The detection method is non-invasive and safe, the operation is simple and convenient, requiring only a small amount of blood to complete the detection, and the sample can be repeatedly collected. CfDNA/CtDNA can carry the same genetic changes and epigenetic information as tumor issues [9], such as point mutations, copy number variations, promoter methylation, microsatellite instability, and loss of heterozygosity. It can overcome tumor heterogeneity [10], reflect the tumor load of human body [11], and then dynamically and timely reflect the patients' conditions. These features make cfDNA/ctDNA a promising biomarker.

## 2. CfDNA/CtDNA

### 2.1. The Development History of cfDNA/ctDNA

**Figure 1:**
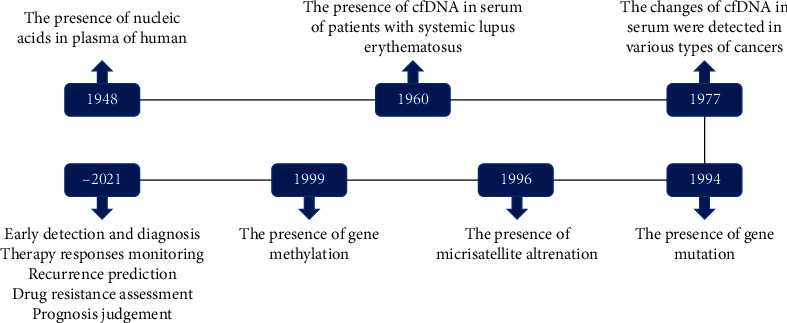
The development history of cfDNA/ctDNA is detected in many diseases and cancers. The detection can involve the concentration and integrity, mutation, methylation of cfDNA/ctDNA, and so on. Analyses of cfDNA/ctDNA can be used to early detection and diagnosis, therapy responses monitoring, recurrence prediction, drug resistance assessment, and prognosis judgment.

CfDNA is a kind of free DNA that exists outside the cells and can be detected in blood, urine, and other body fluids. Mendel and Metais [12] first discovered the presence of cfDNA in human blood in 1948. About 20 years later, Tan [13] detected cfDNA in the serum of patients with systemic lupus erythematosus. Then, Leon [14] found the changes of cfDNA are reflected in various types of cancer. The levels of cfDNA increased in the serum of cancer patients and then decreased after treatment. If the levels of cfDNA remained high, it might indicate a lack of response to chemotherapy. And the increasing levels of cfDNA might be a sign of recurrence of tumor or poor prognosis. Since then, there were more and more studies about cfDNA. In 1994, the N-ras gene point mutation was confirmed in cfDNA of patients with myelodysplastic syndrome or acute myelogenous leukemia [15]. Subsequently, it was reported that microsatellite alterations [16, 17] and gene methylation [18, 19] were also presented in cfDNA from cancer patients, and cfDNA could be used as a marker for early diagnosis, evaluation of cancer therapeutic effect, and judgment of prognosis (Figure 1).

### 2.2. The Sources and Characteristics of cfDNA/ctDNA

**Figure 2:**
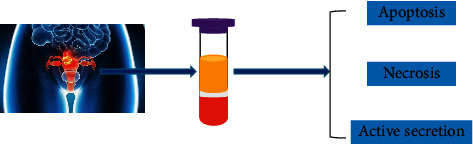
The sources of cfDNA/ctDNA. The circulating DNA in the blood stream comes from tumour cells, which may have mechanisms (apoptosis, necrosis, and active secretion).

For healthy individuals, circulating DNA in plasma comes from apoptotic cells [20]. Circulating DNA is released through physiological processes and may be cleared by its own system; for instance, macrophages in the blood remove free material from damaged or dead cells, which is normal metabolism. When tumor occurs, somatic cell apoptosis is also a source of circulating DNA, because the trapezoidal pattern of plasma or serum DNA is similar to that of apoptotic cells [21]. Beside the DNA release by apoptotic tumors cells, it also includes the DNA release by necrotic tumor cells; as a result, necrosis is an important cause of the presence of DNA fragments. Similarly, the tumor cells secretion can also release DNA [22]. Only a small part of circulating DNA from tumor cells, thus, is called ctDNA. In addition, fetal DNA fragment released into the maternal circulation during pregnancy is the source of cfDNA [23]. And cfDNA may be derived from leukolysis, infection, trauma, and empyrosis [24, 25]. The exact mechanism of cfDNA released from cells into circulation is still unclear, but it is certain that cfDNA cannot be a single source, but multiple sources. CfDNA from different sources can interact with each other, creating cascades that releasing DNA into the loop (Figure 2).

Agarose gel electrophoresis showed that the purified DNA in plasma was double-stranded DNA and composed fragments can be up to 21 kb [26]. The concentration of circulating DNA in plasma from healthy people is very low, 6.6–5.0 ng/ml, and the average length of cfDNA is 176 bp [20]. But the concentration of circulating DNA is significantly increased in malignant tumors and moderately increased in benign diseases [27]. The plasma DNA fragments of cancer patients are longer than those of noncancer patients [28]. However, the length of ctDNA is shorter than cfDNA (133–144bp vs 167 bp) [29]. The difference of cfDNA levels may be related to tumor type, stage, tumor load, and other factors [30]. In order to better apply cfDNA/ctDNA to clinical practice, the biological characteristics of them still need to be continuously explored. They provide a sufficient and powerful basis for the follow-up research and contribute to the study of its clinical application.

### 2.3. The Clinical Applications of cfDNA/ctDNA in Ovarian Cancer

CfDNA/CtDNA plays an important role in ovarian cancer management; hence, we use PubMed database to collect relevant articles about the clinical applications of cfDNA/ctDNA in ovarian cancer. An overview of the research studies on ctDNA/ctDNA in ovarian cancer is summarized (Table 1).

### 2.4. The Diagnostic Value of cfDNA/ctDNA in Ovarian Cancer

In recent years, many researchers have studied the value of cfDNA in the early detection and diagnosis of ovarian cancer. For example, Shao et al. [31] found the levels of cfDNA in the ovarian cancer group were significantly higher than those in the benign ovarian disease group and the healthy control group. It was in accordance with result of Kamat AA′ research [32]. Shao et al. [31] also found that levels of cfDNA were significantly increased in ovarian cancer patients with stage 3–4 compared with those in ovarian cancer patients with stage 1–2. The area under the receiver operating characteristic (ROC) curve was 0.917, and the sensitivity and specific were 88.9% and 89.5%, respectively. The detection of cfDNA was more sensitive and specific than traditional tumor markers, and the diagnostic performance can be further improved when combined detection of these biomarkers. Capizzi et al [33] found the quantitative detection of cfDNA can separate malignant ovarian cancer from benign ovarian disease and healthy people with 77% sensitivity and 96% specificity. Pereira et al. [34] found ctDNA was detected in 93.8% of ovarian cancer patients and significantly correlated with serum CA125 and computed tomography (CT) examination. But detection of ctDNA was more sensitive. However, Zhou meta-analysis found that even though the quantitative detection of cfDNA had a high specificity of 0.90, its sensitivity was low to 0.70 [35]. In conclusion, the quantitative analysis of cfDNA has unsatisfactory sensitivity but acceptable specificity for the diagnosis of ovarian cancer. Stamenkovic experiment [36] found that the correlated co-efficiency between the values of cfDNA concentration and cfDNA integrity were 0.86 and 0.71. The area under curve (AUC) of cfDNA concentration was 0.81, and the AUC of cfDNA integrity was 0.60. However, the AUC of combined detection was 0.84, achieving the best diagnostic effect. Similarly, Yu experiment [37] found that diagnostic value of AUC for cfDNA concentration was 0.86 and for cfDNA integrity was 0.72. When combined detection of them, the diagnostic value was 0.90. According, the joint effect of diagnosis is superior to single detection; the combined testing of cfDNA concentration and cfDNA integrity was favorable to diagnosis for ovarian cancer. The reasons for the differences of the experimental results might be related to a variety of factors. Only when a uniform standard is achieved can cfDNA/ctDNA be better applied to clinical practice. Consequently, further studies are needed to analyze the factors that may influence the diagnostic sensitivity and specificity of ovarian cancer and to validate the diagnostic efficiency of using cfDNA alone or in combination with traditional methods.

TP53 mutation is the most common in high-grade serous ovarian cancer, accounting for more than 95% of somatic mutations [70]. Detection of TP53 mutations in cfDNA/ctDNA has been reported [38–40]. The studies showed that there were the same TP53 mutations in ovarian cancer tissues and matched blood samples. Tumor-derived DNA mutations could be detected in the plasma of some ovarian cancer patients, especially those with advanced ovarian cancer patients. Therefore, detection of TP53 mutations in cfDNA/ctDNA could assist in the diagnosis of ovarian cancer and determine the malignant degree of ovarian cancer. However, the diagnostic performance has not been reported; different methods and different detection techniques lead to different results. It is needed to test the sensitivity and specificity about diagnosis of ovarian cancer. Meanwhile, it is needed to determine whether other ovarian cancer-related mutations are appropriate for diagnosis. The studies of gene mutations have great potential for diagnosing ovarian cancer.

Changes of DNA methylation have been revealed to be an early event in tumorigenesis [71]. Circulating DNA methylation may be a potential marker for early diagnosis of the ovarian cancer [41]. There were several methylation changes in tissues and corresponding plasma samples of ovarian cancer. For example, Dvorská *D* et al. [42] showed that, in the tissues of malignant ovarian cancer patients, the methylation levels of CDH1 gene were higher than those of healthy controls, and the difference was statistically significant. Methylation of CDH1 gene was also highly expressed in the corresponding plasma samples. Wu et al. [43] verified that abnormal methylation of RASSF2A has a frequency of 51.1% in the tissues of patients with epithelial ovarian cancer and 36% in plasma, but has not been detected in benign tumors or healthy individuals. Swamy et al. [44] detected the hypermethylation of RASSF1A and BRCA1 in ctDNA of ovarian carcinoma. The rates of methylation were 31.9% and 56.9%, respectively. This suggested that the methylation pattern of gene in tumor tissues DNA is similar to that in cfDNA/ctDNA of ovarian cancer; aberrant methylation of cfDNA/ctDNA may be valuable markers in ovarian cancer. Furthermore, compared with quantitative detection, qualitative detection of DNA methylation has better diagnostic value [45]. Wang et al. [46] showed no significant difference in CA125 level between patients with early epithelial ovarian cancer and healthy controls by one-way ANOVA analysis. However, the OPCML methylation level of cfDNA was significantly different in early epithelial ovarian cancer patients compared with healthy controls. Hence, this supports the idea that specific methylation could identify epithelial ovarian cancer from healthy individuals and the detection of cfDNA methylation was more sensitive and specific than traditional markers. Liggett et al. [47] found methylation differences of cfDNA in RASSF1A, CALCA, and EP300 could distinguish malignant ovarian tumors from control group, with a sensitivity of 90.0% and specificity of 86.7%. Widschwendter et al. [48] revealed that the methylation pattern of ctDNA, which distinguished high-grade serous ovarian cancer patients from benign patients and healthy women, had a sensitivity of 41.4% and a specificity of 90.7%. Thus, abnormal methylation of cfDNA/ctDNA can be used to early diagnose ovarian cancer, which has good prospects for clinical application. But, the sensitivity and sensitivity of diagnosis are different. Analyzing the causes of the differences is helpful to improve the diagnostic efficiency; it is needed to further study and confirm the diagnostic value of cfDNA methylation.

Chromosomal instability is also an important sign in ovarian cancer and can be detected in cfDNA. Although there were few reports about chromosome instability, preliminary study has shown that it is useful for the diagnosis of ovarian cancer and has potential in clinical research. Vanderstichele et al. [49] demonstrated that the measurements of chromosomal instability in cfDNA from ovarian cancer patients were highest, compared to the benign patients and healthy controls. Especially in high-grade serous ovarian cancer, the AUC of cfDNA detection was 0.94, the specificity was 99.6%, and the sensitivity was 2–5 times higher than that of CA125 and malignant index risk. Thus, chromosomal instability in cfDNA can be suitable for diagnosis of ovarian cancer with high sensitivity and specificity.

### 2.5. The Monitering Value of cfDNA/ctDNA in Ovarian Cancer

#### 2.5.1. Response to Therapy

As a widely used of marker during treatment and follow-up, CA125 performed poorly in clinical application [72]. Conversely, it was reported that cfDNA/ctDNA might play an important role in reflecting therapeutic response of cancer patients. Shao et al. found the levels of cfDNA increased significantly the first day after surgery, but as time went on, the levels of cfDNA gradually declined [31]. Cheng et al. [50] showed that, during the first and second weeks of radiation therapy, the levels of cfDNA in eleven of cancer patients increased eightfold over a period of time, and then decreased at the end of the treatment. However, the levels of cfDNA in the other two cancer patients decreased during treatment. Capizzi et al. [34] verified that cfDNA levels could significantly differentiate between before and after chemotherapy of ovarian cancer patients, which was related to the situation of patients after chemotherapy. Kamat et al. [51] thought that the levels of cfDNA increase is related to the apoptosis index of tumor cells. However, as the DNA was quickly cleared, cfDNA gradually declined. It showed that the levels of cfDNA were significantly associated with tumor burden. As tumor burden increased, so did cfDNA. In a word, the concentration of cfDNA increased in cancer patients and decreased after effective treatment. The variations of cfDNA concentration in cancer patients can dynamically reflect the development and progression of ovarian cancer. The changes of cfDNA levels have a statistically significant correlation with the response to treatment, but correlation was not demonstrated with carcinoma antigen 15–3 (CA15-3), carcinoma antigen 19–9 (CA19-9) [52]. Similarly, ctDNA could also be applied to evaluate treatment response dynamically [35], because the concentration of ctDNA cannot be detected after six months of initial treatment. It suggested that patients might respond well to treatment. Accordingly, cfDNA/ctDNA could serve as a meaningful biomarker to monitor disease progression and therapeutic response, meanwhile, becoming a tool for reflecting tumor load. Monitoring changes of cfDNA levels may have benefit for the ovarian cancer patients.

Analyzing status of gene mutations and methylation changes in cfDNA/ctDNA is also helpful for understanding patients' respond to treatment. Arend et al [53] indicated that 38 genetic variations were detected in six genes in tumor DNA before the neoadjuvant chemotherapy. And there were 59 mutations in the nineteen genes in cfDNA. After the neoadjuvant chemotherapy, 33 of the 38 variations in tumor DNA remained unchanged, while only 6 of the 59 mutations were present in cfDNA. Therefore, detection of cfDNA gene variations may better reflect the response to chemotherapy in patients with high-grade serous ovarian cancer. But, this still requires a larger number of cases to expand the tests and determine the role of cfDNA mutations in ovarian carcinoma. TP53 mutation is a characteristic marker for high-grade serous ovarian cancer and might reflect the conditions of patients. After chemotherapy, TP53 mutations in serum ctDNA were not detect, but reappeared as the disease progresses [54]. Kim YM's experimental results manifested that TP53 mutant allele fraction in ctDNA significantly decreased after therapy, and no significant difference in the rate of descent compared with CA125 [55]. However, the result of Parkinson CA′ research demonstrated that ctDNA responded to treatment earlier than CA125. And patients with TP53 mutation allele fraction in ctDNA decreased by less than 60% were associated with adverse reactions [56]. Therefore, TP53 mutations in ctDNA may be a potential marker to monitor therapeutic response in ovarian cancer, and have crucial research value. After chemotherapy, methylation levels of ctDNA decreased significantly [47]. After surgery, specific chromosomal rearrangements in cfDNA were not detected in 5/8 patients [57], which suggests a good response to treatment. Therefore, methylation changes and specific chromosomal rearrangements might play an important role in reflecting the therapeutic effect. They had potential in monitoring the disease progression. But, because of the lack of research, there are few reports about their response for ovarian cancer treatment; the role of them should be further demonstrated by a large number of experiments. As a consequence, the analysis of cfDNA/ctDNA can assess the tumor load and better reflect the response to treatment, so as to make a treatment plan and provide reference for subsequent treatment. Current studies support the increasing important value of cfDNA/ctDNA as a new monitoring tool for patients during therapy.

#### 2.5.2. Recurrence and Metastasis

Although most ovarian cancer patients have good respond to treatment, advanced ovarian cancer patients tend to relapse after 1 to 2 years of treatment. It is related to the patients' age, histological type, tumor stage, and other factors. And ovarian cancer is prone to metastasis; 70% malignant tumors spread to pelvic and abdominal organs. The evaluation of recurrence and metastasis mainly relies on CA125 and CT, but CA125 and CT cannot monitor dynamically and timely the situation of ovarian cancer patients after recurrence, and the detection of metastatic lesions is also limited. However, the use of cfDNA is promising for monitoring the recurrence and metastasis in ovarian cancer patients. During tumor recurrence, the levels of PIK3A-H1047 R in cfDNA increased again, and it had a correlation with metastasis [58]. Parkinson et al. indicated that patients with relapsed ovarian cancer have higher levels of ctDNA than those with newly diagnosed ovarian cancer patients [56]. Vitale et al. [54] demonstrated that TP53 mutation was present in the serum circulating cell-free tumor DNA of relapsed high-grade serous ovarian cancer patients. After chemotherapy, TP53 mutation reduced to undetectable level in ctDNA, but increased again as the disease progressed, TP53 mutation can be used as an indicator of disease monitoring and to judge recurrence. When patients with high-grade serous ovarian cancer recurred, an unbiased analysis of cfDNA could detect the BRCA1/2 reversion mutations [59]. Gifford et al. [73] expound that hMLH1 methylation increased in the plasma DNA after chemotherapy, which indicated that the ovarian cancer patients relapsed. Hence, the changes of cfDNA can reflect the situation of ovarian cancer patients. In summary, the detection of cfDNA/ctDNA concentration is helpful for the monitoring of metastasis and recurrence of tumor, and the gene mutations and methylation changes of cfDNA/ctDNA also have great significance for development and progress of tumor. Monitoring the changes of cfDNA/ctDNA is positive to ovarian cancer.

#### 2.5.3. Resistance to Chemotherapy

Resistance to chemotherapy is common among patients during the development and progression of the diseases; cfDNA/ctDNA in the treatment of chemotherapy resistant ovarian cancer has an important effect. Steffensen et al. [60] proved that the use of bevacizumab contributes to treatment of multi-resistance epithelial ovarian cancer. Depending on the levels of cfDNA, treatment could be guided; it could be applied as an assistive marker. The BRCA1/2 mutations could be detected in the ctDNA from ovarian cancer patients, which responded well to the targeted therapy of the PARP1 inhibitors [61]. The study about the BRCA1/2 mutations is a breakthrough and provides a better insight into response to chemotherapy. But reversion mutations tend to lead to a high incidence of clinically acquired drug resistance. The BRCA1/BRCA2 reversion mutations in cfDNA were found by sequencing analysis from 21% of therapy-resistant of ovarian cancer patients [62]. The acquisition of BRCA1/2 reversion mutations was closely related to resistance to therapy and may be beneficial to predict the chemotherapy response of ovarian cancer, guiding the treatment of ovarian cancer. However, its specific mechanism is unclear. It is needed to further study and verify the role of BRCA1/BRCA2 reversion mutations in ovarian cancer.

### 2.6. The Prognostic Value of cfDNA/ctDNA in Ovarian Cancer

Ovarian cancer patients had a poor overall prognosis. Despite the fact that there were major breakthroughs in surgery and chemotherapy, the survival of ovarian cancer patients did not improve significantly. 5-year survival rate of advanced ovarian cancer patients was significantly lower than that of early ovarian cancer patients. Therefore, tumor markers are urgently needed to assess the prognosis of ovarian cancer patients. Quantitative analysis of cfDNA/ctDNA is reported to be beneficial in evaluating the prognosis of ovarian cancer. When the levels of cfDNA exceed a certain range, the risk of death increases, which is related to the decreased survival rate of ovarian cancer patients [63]. The concentration of RAB25 in cfDNA was correlated with overall survival and progression-free survival. The low levels of RAB25 predicted better PFS and OS [64]; it was a prognostic indicator for epithelial ovarian cancer. CfDNA also showed prognostic importance for chemoresistant ovarian cancer patients. Patients with high levels of cfDNA had poor PFS and OS [60]. Hence, monitoring the changes of cfDNA levels can help adjust therapeutic regimens and observe the state of ovarian cancer patients.

Detection of mutations in cfDNA/ctDNA also has important value for the prognosis of ovarian cancer. The frequency of somatic mutations in plasma from patients with stage 1 or 2 ovarian cancer was 68%. As the tumor stage increased, so did the mutant allele fraction in ctDNA. Patients with high ctDNA levels had poor PFS and OS [65]. One-third of ovarian cancer patients have tumor-specific TP53 mutation in plasma, which have low survival rate. Circulating tumor DNA was an independent predictor of low survival in multivariate analysis [40]. Serous ovarian cancer patients with TP53 antibodies had poor overall survival [66]. Meanwhile, the TP53 mutation in ctDNA from high-grade serous ovarian cancer patients is associated with stage. Three months after chemotherapy, the high TP53 mutation allele fraction in ctDNA indicated the poor progression [55]. There was a more significant prognostic effect than CA125. Hence, detection of TP53 mutation in cfDNA/ctDNA is valuable for judging prognosis of ovarian cancer. In addition, analysis of TP53 mutation in the plasma DNA can determine the degree of malignant ovarian cancer and is helpful for postoperative follow-up [38]. The frequency of KRAS mutation was particularly high in ovarian mucinous carcinoma, and KRAS mutation was associated with poor overall survival [66]. The meta-analysis clarified the presence of KRAS mutation in epithelial ovarian cancer, and the KRAS mutation in cfDNA was associated not only with poor OS but also with poor PFS [67]. So, the detection of KRAS mutation in cfDNA was beneficial to the prognosis of ovarian cancer patients. Then, the researchers detected PI3CA and KRAS mutations in cfDNA from ovarian clear cell carcinoma using ddPCR and found that patients with higher levels of PIK3CA-H1047 R and KRAS-G12D had shorter PFS [58]. The changes of two indexes were more sensitive and rapid than CA125. Consequently, assessing the status of mutations may provide important information for the prognosis of patients with ovarian cancer.

In high-grade serous ovarian cancer patients, there was ERS1 methylation in primary tumors and paired circulating tumor DNA, and ESR1 methylation had a remarkable consistency between primary tumors and paired circulating tumor DNA. The presence of ESR1 methylation in primary tumors was associated with better OS, PFS, and clinicopathologic features, such as age and tumor rest; however, there was no correlation in ctDNA [68]. RASSF1A promoter methylation also was found in high-grade serous ovarian cancer patients. The levels of RASSF1A promoter methylation in primary tumors were higher than those in adjacent morphologically tumor cell-free tissues, and RASSF1A promoter methylation was also detected in paired circulating tumor DNA. RASSF1A promoter methylation in primary tumors was related to tumor grade and regional lymph node metastasis. Moreover, RASSF1A promoter methylation was positively associated with OS. Nevertheless, there was no significant correlation between RASSF1A promoter methylation and clinicopathological characteristics or OS in adjacent tissues and paired plasma samples [69]. Although methylation can be detected in ctDNA, the role of methylation in ctDNA is unclear. Further researches are needed to understand whether cfDNA/tDNA can predict disease outcomes and evaluate the prognosis of ovarian cancer. Subsequently, the studies showed that methylation of RASSF1A and BRCA1 was evident in different stages and grades of ovarian cancer and might have potential as a prognostic marker in ovarian cancer patients. The presence of hMLH1 methylation in plasma DNA from relapsed ovarian cancer patients was associated with poor OS and was independent of age, disease duration, and other factors. So, the changes of DNA methylation in cfDNA provided potential for prognosis of patients with ovarian cancer [73]. There are few studies about the prognostic value of cfDNA/ctDNA methylation in ovarian cancer, and the mechanism by which methylation occurs in the blood is unclear. In a word, further efforts are needed to screen specific methylation and confirm the significance of cfDNA/ctDNA methylation in prognosis of ovarian cancer.

## 3. Future Directions and Challenges

With the continuous development and innovation of technology, cfDNA has become a research focus in the medical field and has a broad application prospect. CfDNA/ctDNA has obvious advantages over traditional methods. It can not only be used for prenatal screening [74], analysis of immune diseases [75, 76], but also have very important clinical value in oncology. It has been reported in colorectal cancer, breast cancer, non-small cell lung cancer, and other tumors [77–82]. The value of cfDNA/ctDNA can be demonstrated and utilized through diagnosis, monitoring of therapeutic response, recurrence and drug resistance, and prognosis. CfDNA/ctDNA is a prospective marker that provides important evidence for clinical research and application.

Although the development of “liquid biopsy” has made tremendous progress, it still faces many challenges. If cfDNA/ctDNA is to be used effectively in the clinic, there are some problems to be solved. For instance, the exact source and mechanism of cfDNA/ctDNA are unclear, which will affect subsequent research. The problems of collection and treatment of samples, extraction of cfDNA/ctDNA, and analysis of outcome will also interfere with the results of the experiment. Different experimental subjects were selected and different test methods and techniques were used, resulting in the different results. The sensitivity and specificity of detection still need to be improved. Therefore, it still needs to make efforts to develop standardized procedures for early application in clinical trials.

## 4. Conclusions

Not only the detection of concentration and integrity but also the genetic mutations and methylation changes have been reported in cfDNA/ctDNA of ovarian cancer. As the new tumor marker, cfDNA/ctDNA plays a key role in the clinical application. It can be used to screen and detect tumors and evaluate prognosis, therapeutic effects, and response to chemotherapeutic drugs. However, the value of cfDNA/ctDNA still needs to be explored continually. In the future, cfDNA/ctDNA has tremendous potential of development and broad prospects of clinical application. Further efforts are needed to bring cfDNA/ctDNA into clinical practice at an early date.

## Figures and Tables

**Table 1:** An overview of the research studies on ctDNA/ctDNA in ovarian cancer is summarized.

Author	Year	Application	Sample	Method	Target
Shao et al. [31]	2015	Diagnosis	Serum	bDNA technique	The levels of cfDNA
Kamat et al. [32]	2006	Diagnosis	Plasma	Real-time PCR	The levels of cfDNA
Capizzi et al. [33]	2008	Diagnosis/therapy response	Plasma	Real-time PCR	The levels of cfDNA
Pereira et al. [34]	2015	Diagnosis	Serum	Droplet digital PCR	The levels of ctDNA
Zhou et al. [35]	2016	Diagnosis	Peripheral blood	Quantitative real-time PCR	The levels and integrity of cfDNA
Stamenkovic et al. [36]	2020	Diagnosis	Peripheral blood	Quantitative real-time PCR	The levels and integrity of cfDNA
Yu et al. [37]	2019	Diagnosis	Peripheral blood	Quantitative real-time PCR	The levels and integrity of cfDNA
Otsukaet al. [38]	2004	Diagnosis	Plasma	F-SSCP	TP53 mutations
Park et al. [39]	2018	Diagnosis	Plasma	Digital PCR	TP53 mutations
Swisher et al. [40]	2005	Diagnosis	Peripheral blood	Ligase detection reaction	TP53 mutations
Battagli et al. [41]	2004	Diagnosis	Peripheral blood	MSP	BRCA1 and RASSF1A methylation
Dvorská et al. [42]	2019	Diagnosis	Plasma	MSP	Gene methylation
Wu et al. [43]	2014	Diagnosis	Serum	MSP	RASSFA methylation
Sandeep et al. [44]	2019	Diagnosis	Plasma	MSP	RASS1A and BRCA1 methylation
Li et al. [45]	2019	Diagnosis	Peripheral blood	NGS	Qualitative detection (methylation)
Wang et al. [46]	2017	Diagnosis	Serum	MSP	OPCML methylation
Liggett et al. [47]	2011	Diagnosis	Serum	NGS	DNA methylation
Widschwendter et al. [48]	2017	Diagnosis	Serum	Bisulfite sequencing	DNA methylation
Vanderstichele [49]	2017	Diagnosis	Plasma	Low-coverage whole-genome sequencing	Chromosomal instability
Cheng et al. [50]	2009	Therapy response	Plasma	Quantitative PCR	The levels of cfDNA
Kamat et al. [51]	2006	Therapy response	Plasma	Real-time PCR	The levels of cfDNA
Hufnagl et al. [52]	2020	Therapy response	Plasma	Quantitative RT-PCR	The levels of cfDNA
Arend et al. [53]	2018	Therapy response	Plasma	NGS	Mutations
Vitale et al. [54]	2020	Therapy response	Serum	NGS	TP53 mutations
Kim et al. [55]	2019	Therapy response	Plasma	Digital PCR	TP53 mutations
Parkinson et al. [56]	2016	Therapy response	Plasma	Microfluidic digital PCR	TP53 mutations
Harris et al. [57]	2016	Therapy response	Plasma	Quantitative PCR	Chromosomal rearrangements
Morikawa et al. [58]	2018	Therapy response	Plasma	Droplet digital PCR	PIK3CA and KRAS mutations
Christie et al. [59]	2017	Therapy response	Plasma	NGS	BRCA1/2 germline mutations
Steffensen et al. [60]	2014	Therapy response/prognosis	Plasma	Real-time PCR	The levels of cfDNA
Ratajska et al. [61]	2017	Therapy response	Plasma	NGS	BRCA1/2 mutations
Weigelt et al. [62]	2017	Therapy response	Plasma	NGS	BRCA1 and BRCA2 reversion mutations
Kamat et al. [63]	2010	Prognosis	Plasma	Real-time PCR	The levels of cfDNA
No et al. [64]	2012	Prognosis	Serum	Quantitative real-time PCR	The levels of cfDNA
Phallen et al. [65]	2017	Prognosis	Plasma	TES-seq	Genomic mutations
Dobrzyckaet al. [66]	2011	Prognosis	Plasma	PCR-RFLP	KRAS and TP53 mutations
Zhuang et al. [67]	2017	Prognosis	Peripheral blood	Meta-analysis	KRAS mutation
Giannopoulou et al. [68]	2018	Prognosis	Plasma	Real-time MSP	ESR1 methylation
Giannopoulou et al. [69]	2017	Prognosis	Plasma	Real-time MSP	RASSF1A methylation
